# Contribution of eDNA Metabarcoding to Model Ecosystems: Case Study of Threatened Peri-Urban Mediterranean River

**DOI:** 10.3390/biology15201874

**Published:** 2026-10-17

**Authors:** Rosi De Luca, Mirella Vazzana, Mario Lo Valvo, Aiti Vizzini, Slobodanka Radovic, Grazia Orecchio, Angela Cuttitta, Marilena Di Natale, Francesco Longo, Chiara Martino, Vinicius Queiroz, Manuela Mauro

**Affiliations:** 1 Dipartimento di Scienze e Tecnologie Biologiche, Chimiche e Farmaceutiche (STEBICEF), Università Degli Studi di Palermo, Via Archirafi, 18, 90123 Palermo, Italy; rosi.deluca@unipa.it (R.D.L.); mirella.vazzana@unipa.it (M.V.); aiti.vizzini@unipa.it (A.V.); orecchio.grazia@gmail.com (G.O.); francesco.longo03@unipa.it (F.L.); chiara.martino@unipa.it (C.M.); 2 IGA Technology Services Srl., Via Linussio, 51, 33100 Udine, Italy; sradovic@igatechnology.com; 3 Institute for Studies on the Mediterranean (CNR-ISMed), National Research Council, Via Filippo Parlatore, 65, 90145 Palermo, Italy; angela.cuttitta@ismed.cnr.it (A.C.); marilena.dinatale@ismed.cnr.it (M.D.N.); 4 Departamento de Fisiologia, Instituto de Biociências, Universidade de São Paulo, Rua do Matão, Trav. 14, n◦101, São Paulo 05508-090, SP, Brazil; viqueiroz@usp.br

**Keywords:** 12S, biomonitoring, biodiversity, lotic ecosystems, metabarcoding, Sicily

## Abstract

Freshwater ecosystems on Mediterranean islands are threatened refuges where conventional bioassessments often fail to capture the full spectrum of biotic assemblages. Here, environmental DNA (eDNA) metabarcoding targeting the mitochondrial 12S rRNA gene was applied across three representative sampling reaches (Upstream, Midstream, Downstream) of the Oreto River (Sicily, Italy) during a single sampling campaign to assess vertebrate composition. This targeted spatial snapshot captured aquatic, semi-aquatic, and terrestrial signals. Upstream reaches featured synanthropic taxa, while midstream sections showed high signals of livestock (e.g., *Bos taurus*, *Ovis aries*, *Sus scrofa*), likely reflecting agricultural land use. Downstream reaches exhibited high taxonomic heterogeneity, comprising native fauna—including the Critically Endangered European eel (*Anguilla anguilla*)—alongside wild birds, amphibians, and non-native species (e.g., *Ameiurus melas*, *Micropterus salmoides*). By recovering conservation-priority taxa, non-native species, and broader potential anthropogenic-associated signals (including dietary marine fish detections), this study demonstrates eDNA’s effectiveness in capturing complex biological footprints. Although physicochemical conditions were not directly assessed, and despite the temporal limitations of a single snapshot, these findings highlight the promise of eDNA metabarcoding as a complementary tool for future environmental tracking, watershed management, and spatial conservation planning.

## Simple Summary

Freshwater ecosystems on Mediterranean islands host vital wildlife but face growing threats from climate change and human activities. Conventional monitoring methods are often stressful to wildlife, labor-intensive, and miss elusive species. Our study surveyed vertebrates across three representative sampling stations along Sicily’s Oreto River using environmental genetic material continuously shed into the water. While constrained by limited spatial and temporal replication, analyzing water samples provided a spatial snapshot of biological diversity. The results revealed distinct community compositions: upper sections showed synanthropic wildlife signals, middle stretches were characterized by a high proportion of farm animal signals that indicate potential agricultural land use, and lower reaches yielded genetic signatures of endangered native species alongside alien species. Additionally, marine fish DNA in all the sites suggested potential anthropogenic inputs, such as food waste or municipal effluents. In conclusion, collecting waterborne genetic signals provides a fast, non-invasive, and effective way to characterize aquatic biological assemblages. While direct water quality parameters were not measured, this approach offers a practical complementary tool to support biodiversity monitoring, potentially manage land use responsibly, and inform local conservation priorities.

## 1. Introduction

Biodiversity characterization is fundamental for understanding ecosystem dynamics and supporting effective conservation and restoration strategies [1,2]. The urgency of acquiring such information is particularly evident in freshwater ecosystems—comprising rivers, lakes, and wetlands—which, despite covering less than 1% of the Earth’s surface, harbor nearly one-third of all vertebrate species and rank among the most threatened biomes globally due to combined anthropogenic pressures and climate change [3,4,5,6,7]. This vulnerability is especially pronounced in Mediterranean freshwater habitats, where habitat fragmentation, hydromorphological alterations, pollution, invasive alien species (IAS), and, crucially, climate-induced aridification act synergistically to threaten biodiversity [8,9].

Biodiversity assessment in freshwater ecosystems has traditionally relied on field-based surveys, whose effectiveness can vary with habitat characteristics, target taxa, and sampling effort [10,11]. Over the last decade, environmental DNA (eDNA) metabarcoding has become a widely used approach for characterizing aquatic communities from environmental samples [12,13,14,15]. Indeed, although eDNA surveys face recognized methodological constraints—such as variation in DNA shedding, PCR bias, and reference database completeness [16,17,18,19]—ongoing methodological and analytical refinements have consolidated their role as an important component of modern aquatic biomonitoring. This approach demonstrates particular potential in lotic systems, where the hydrographic network functions as a ‘natural collector’, gathering and transporting eDNA shed not only by strictly aquatic species, but also by semi-aquatic and terrestrial fauna interacting with the watershed [20,21,22,23]. Although this integrated landscape-scale tracking has been increasingly demonstrated in several continental European rivers [24,25], its application in insular freshwater systems of the Mediterranean remains relatively limited. These systems often combine strong seasonal variability, habitat fragmentation, and intense anthropogenic pressures, highlighting the need for baseline biodiversity assessments across spatially heterogeneous river reaches. A prime example of such a neglected, highly stressed insular ecosystem is the Oreto River (Sicily, Italy). Serving as a relict of the historical wetland systems of the Palermo plain, the river suffers from chronic anthropogenic stressors, including unauthorized wastewater discharges and severe hydromorphological alterations [26]. Despite these severe environmental pressures, the Oreto River exhibits a remarkable latent resilience, maintaining fragmented ecological niches that harbor significant, yet poorly documented, biotic communities. Our recent research has already begun to unveil this hidden diversity; for instance, eDNA metabarcoding was applied to characterize the river’s macroinvertebrate assemblages, establishing a first baseline for its benthic biodiversity [27]. Regarding vertebrate communities, the most recent work to our knowledge is that of Guadagna (2018) [26], which focused specifically on the terminal stretch of the river. This prior study highlighted the ecological instability of the river and emphasized the need for continuous monitoring to identify and protect biological communities of conservation interest.

In this study, we applied 12S eDNA metabarcoding across three representative sampling reaches (upstream, midstream, and downstream) along the Oreto River. Designed as an exploratory baseline snapshot from a single sampling campaign, and acknowledging the inherent limitations of restricted spatial and temporal replication, this study aims to characterize local vertebrate composition and assess potential spatial shifts in community structure across the three sampled reaches.

Through this approach, our analysis captured a broad spectrum of biological signals—encompassing aquatic, semi-aquatic, and terrestrial fauna (including native, invasive alien, and domestic species)—while providing preliminary insights into potential anthropogenic influences (such as livestock presence and putative inputs from food waste or urban discharges). By establishing an initial genetic baseline, this study highlights both the practical utility and the methodological constraints of rapid, single-campaign eDNA surveys for characterizing local biodiversity and providing preliminary spatial insights in threatened Mediterranean peri-urban river systems.

## 2. Materials and Methods

### 2.1. Study Area and Water Sampling

**Figure 1:**
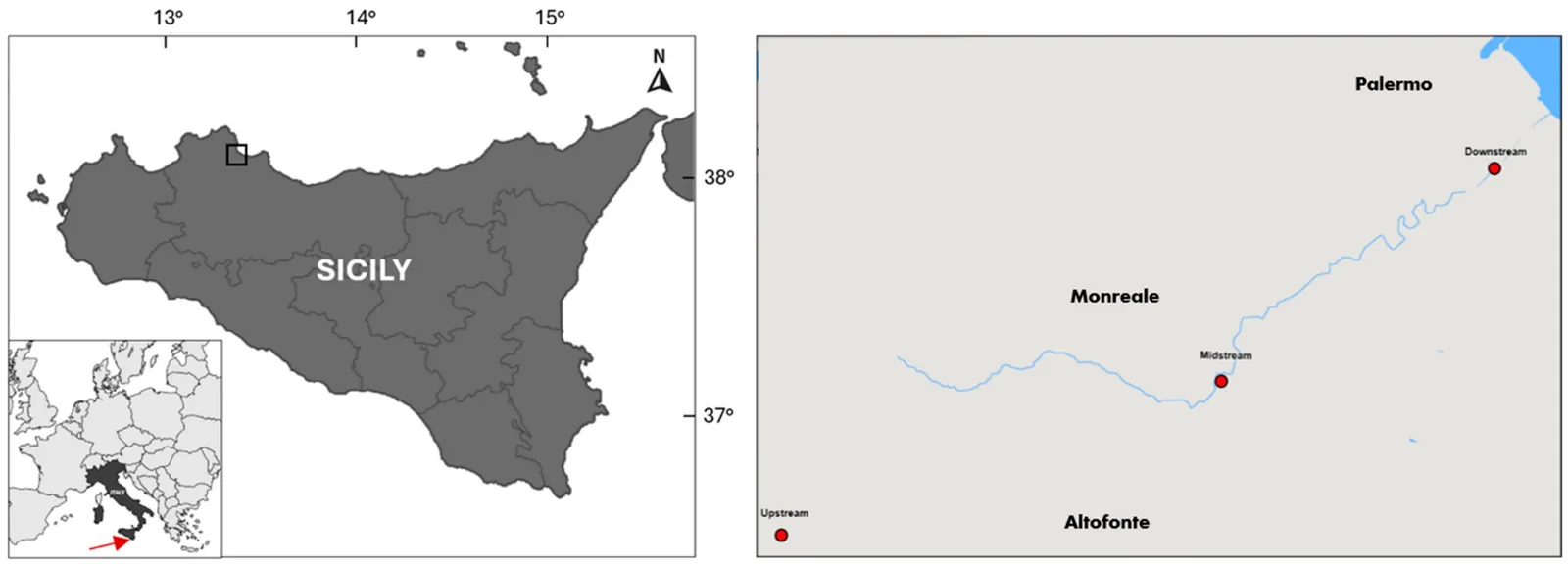
Geographic location of the study area and distribution of the eDNA sampling stations along the Oreto River (Sicily, Italy). The left panel shows the localization of Italy in Europe and of Sicily within the Mediterranean (indicated by the red arrow); the targeted study area is bounded by the black box. The right panel illustrates the spatial distribution of the three selected biological sampling stations (Upstream, Midstream, and Downstream) represented by red dots across the municipalities of Altofonte, Monreale, and Palermo.

This study focused on the Oreto River, a lotic system located in northern Sicily, Italy. The hydrographic basin of the Oreto spans approximately 130 km^2^ and crosses the administrative territories of Altofonte, Monreale, and Palermo [28] (Figure 1). The main stem of the river extends for roughly 20 km with an average annual discharge of 0.86 m^3^/s, geographically bordered by the Jato, Belice, and Eleuterio watersheds to the south-west, south, and east, respectively [29,30,31].

Fieldwork was conducted during a single sampling campaign in October 2023. To assess local variations in vertebrate eDNA signals across three representative sampling locations—chosen based on a regular spatial distribution along the longitudinal gradient, safe operator access, habitat suitability, and sufficient water flow—water samples were collected from three distinct stations: Upstream (38°2′43″ N, 13°13′40″ E; elevation 470 m a.s.l.), Midstream (38°4′13″ N, 13°17′41″ E; elevation 180 m a.s.l.), and Downstream (38°6′9″ N, 13°22′10″ E; elevation 10 m a.s.l.). At each station, three independent biological replicates were gathered to account for local spatial patchiness, yielding a total of nine environmental water samples across the study area (n=9 in total).

For each biological replicate, a 2-L water sample was collected from the mid-depth of the water column using acid-washed (10% HCl) and autoclaved sterile glass bottles. Samples were immediately stored in cooler boxes and transported in darkness to the laboratory for prompt processing.

### 2.2. eDNA Extraction

Upon arrival at the laboratory, water samples were vacuum-filtered under sterile conditions using HC Series filtration systems (Cheimika-HC/SLGS/F05002, Pellezzano, Italy) equipped with 0.22 µm nitrocellulose membrane filters (MF-Millipore, 47 mm MCE, Merck, Darmstadt, Germany). All filtration apparatus—including glass funnels, receiving flasks, tweezers, and scissors—as well as the dedicated workspaces, were rigorously decontaminated with 10% bleach and exposed to UV light for 20 min before each use. To ensure the absolute absence of cross-contamination, negative filtration controls consisting of MilliQ water and extraction blanks were processed alongside the environmental samples. All filtration and extraction controls yielded non-detectable DNA concentrations upon fluorometric quantification (Qubit dsDNA HS Assay; Invitrogen, Thermo Fisher Scientific, Waltham, MA, USA). For the molecular phase, each membrane filter was sectioned into approximately 1 mm strips to maximize lysis efficiency. Total environmental DNA (eDNA) was extracted independently from each membrane filter (yielding nine separate eDNA extracts) using the DNeasy Blood & Tissue Kit (Qiagen, Hilden, Germany) following the manufacturer’s optimized protocol. The resulting eDNA extracts were stored at −20 °C until downstream high-throughput sequencing assays [32].

### 2.3. Amplification and High-Throughput Sequencing

Metabarcoding of the nine independent eDNA extracts was performed by IGA Technology Services s.r.l. (Udine, Italy) using a two-step PCR approach targeting the mitochondrial 12S rRNA gene. To mitigate stochastic PCR effects and amplification biases, the initial PCR step for each of the nine biological extracts was performed in technical triplicates. The three resulting PCR products per extract were subsequently pooled prior to purification and dual-indexing, yielding a single sequencing library per biological replicate. The initial PCR step amplified a 106 bp fragment from the 12S region. Each reaction was performed in a final volume of 25 µL containing: 12.5 µL of 2 × KAPA HiFi HotStart ReadyMix (Roche, Wilmington, MA, USA), 2.5 µL of forward primer 12SV5-F 5′-ACTGGGGATTAGATACCC-3′ (with Illumina adaptor 2 µM; Illumina, San Diego, CA, USA) and 2.5 µL of reverse primer 12SV5-R 5′-TAGAACAGGCTCCTCTAG-3′ (with Illumina adaptor 2 µM; Illumina, San Diego, CA, USA). The mix was then supplemented with 7.5 µL of the DNA extract. The thermal profile consisted of an initial denaturation at 95 °C for 3 min, followed by 30 cycles of denaturation at 95 °C for 30 s, annealing at 55 °C for 30 s, and extension at 72 °C for 30 s, with a final extension at 72 °C for 4 min. Amplified products were purified using 1.6× Ampure XP magnetic beads (Beckman Coulter Life Sciences, Indianapolis, IN, USA) and eluted in 35 µL of Tris-HCl buffer (pH 8.0). In the second PCR step, dual-indexing adapters were attached to the amplicons to allow sample multiplexing. The reactions contained 7.5 µL of the purified first-step PCR product, 12.5 µL of 2× KAPA HiFi HotStart ReadyMix, and 2.5 µL of each Nextera XT index primer (Illumina, San Diego, CA, USA). Additionally, no-template PCR controls (NTCs) were included in all amplification runs. Electrophoretic profiling confirmed that NTCs yielded only low-concentration adapter dimers without target amplicons (~106 bp), and they were therefore excluded from the final sequencing pool. The cycling conditions were: 95 °C for 3 min; 9 cycles of 95 °C for 30 s, 55 °C for 30 s, and 72 °C for 30 s; followed by a final extension at 72 °C for 5 min. The resulting libraries were quantified with the Qubit 1X dsDNA HS Assay Kit (Invitrogen, Thermo Fisher Scientific, Waltham, MA, USA), pooled in equimolar concentrations, and sequenced on an Illumina NovaSeq 6000 2 × 150 bp platform (Illumina, San Diego, CA, USA).

### 2.4. Bioinformatics Processing and Taxonomic Assignment

Metabarcoding sequences were processed using a custom bioinformatic pipeline developed by IGATech (Udine, Italy). When amplicon length allowed, the 3′-ends of paired-end reads were merged using FLASH v. 1.2.11 (--max-overlap 70, --min-overlap 25) to generate consensus pseudo-reads. Only successfully merged consensus reads were retained and utilized for downstream processing and taxonomic assignment. Primer sequences were removed using Cutadapt v.2.7 (--discard-untrimmed, --minimum-length 70, --overlap 10, --times 2, --error-rate 0.15), retaining only reads of at least 70 bp. Low-quality bases at the 3′ ends were trimmed using ERNE-filter v.1.4.3 (--min-size 70). Subsequent analyses were performed within the QIIME v.1.9.1 environment. Putative chimeric sequences were identified using VSEARCH v.2.14.1 and removed prior to taxonomic analysis. Operational Taxonomic Unit (OTU) picking was performed in open-reference mode against the 12S Vertebrate Reference Set for the RDP Classifier (release v2.0.0). This database, structured according to the NCBI taxonomy, contains 19,654 reference sequences representing 15,007 taxa across all taxonomic ranks, including 9564 species, and was compiled from the NCBI nucleotide database (July 2021) and MitoFish (March 2020). During the OTU-picking step, reads were matched against the reference database using the uclust algorithm with a minimum sequence identity threshold of 97%. This threshold was employed exclusively for OTU clustering and reference matching and should not be interpreted as a criterion for species-level taxonomic assignment.

Taxonomic classification of representative OTU sequences was subsequently performed using the Ribosomal Database Project (RDP) Classifier v.2.2 and the taxonomy mapping file associated with the reference database. Taxonomic assignments were retained when the classifier confidence score was ≥0.50, following recommendations for short amplicon datasets. The metabarcoding assay targeted the vertebrate 12S-V5 region and generated amplicons of approximately 106 bp. Given the limited length of the amplified fragment, taxonomic resolution is not uniform across vertebrate groups, and closely related species may share identical or highly similar sequences within the targeted region. Consequently, species-level assignments reported in this study should be considered the highest-resolution classifications supported by the available reference database and classifier output rather than unequivocal species identifications.

To improve transparency, the confidence score associated with each taxonomic assignment is reported in Table S1.

Sequences were subsequently filtered to retain only taxa belonging to the phylum Chordata. Reads assigned to *Homo sapiens* and *Pan troglodytes* were removed as likely laboratory or environmental contaminants. Sequences remaining unclassified below the Order level were excluded from downstream ecological analyses. Raw data and sequence datasets were deposited in GenBank (BioProject n. PRJNA1542808).

### 2.5. Data Analysis

Statistical analyses and data visualizations were performed in R (v. 4.5.1). Bioinformatic processing retained all nine biological replicates (n=9) as independent sample units. To standardize sequence yield variations across samples, raw read counts were converted to relative read abundance (RRA, %).

For station-level taxonomic summaries, reads from the three biological replicates were computationally aggregated per site. Hierarchical flow diagrams (Sankey diagrams) tracking assigned taxa across different taxonomic levels were generated using the open-source web application SankeyMATIC (https://sankeymatic.com, accessed on 16 July 2026). The spatial distribution of the vertebrate community was visualized using a bubble plot generated via the ‘tidyverse’ and ‘ggplot2’ packages; for this specific representation, taxa were grouped a priori into five ecological macro-categories (Wild, Alien, Farm & Domestic, Synanthropic, and Food).

To evaluate spatial variations in vertebrate community composition and beta diversity across the three sampling sites, a Principal Coordinates Analysis (PCoA) was performed on a Bray–Curtis distance matrix using the nine independent biological replicates (n=3 per station). Differences among stations were tested using permutational multivariate analysis of variance (PERMANOVA, 999 permutations), complemented by an analysis of multivariate homogeneity of group dispersions (betadisper) to evaluate within-group variability. Species scores were derived via weighted averages of replicate coordinates to project taxa and samples jointly onto the ordination space, with label positioning optimized using ’ggrepel’.

## 3. Results

The filtering and quality curation process reduced the total number of readings at all three sampling points: from 1,098,128 to 186,334 at the Upstream station, from 2,466,627 to 761,928 at the Midstream station, and from 1,598,622 to 357,649 at the Downstream station. Overall, eDNA metabarcoding analysis revealed a diverse vertebrate assemblage across the sampling sites, encompassing four taxonomic classes (Actinopterygii, Amphibia, Aves, and Mammalia), represented by 25 orders, 35 families and 47 distinct species.

**Figure 2:**
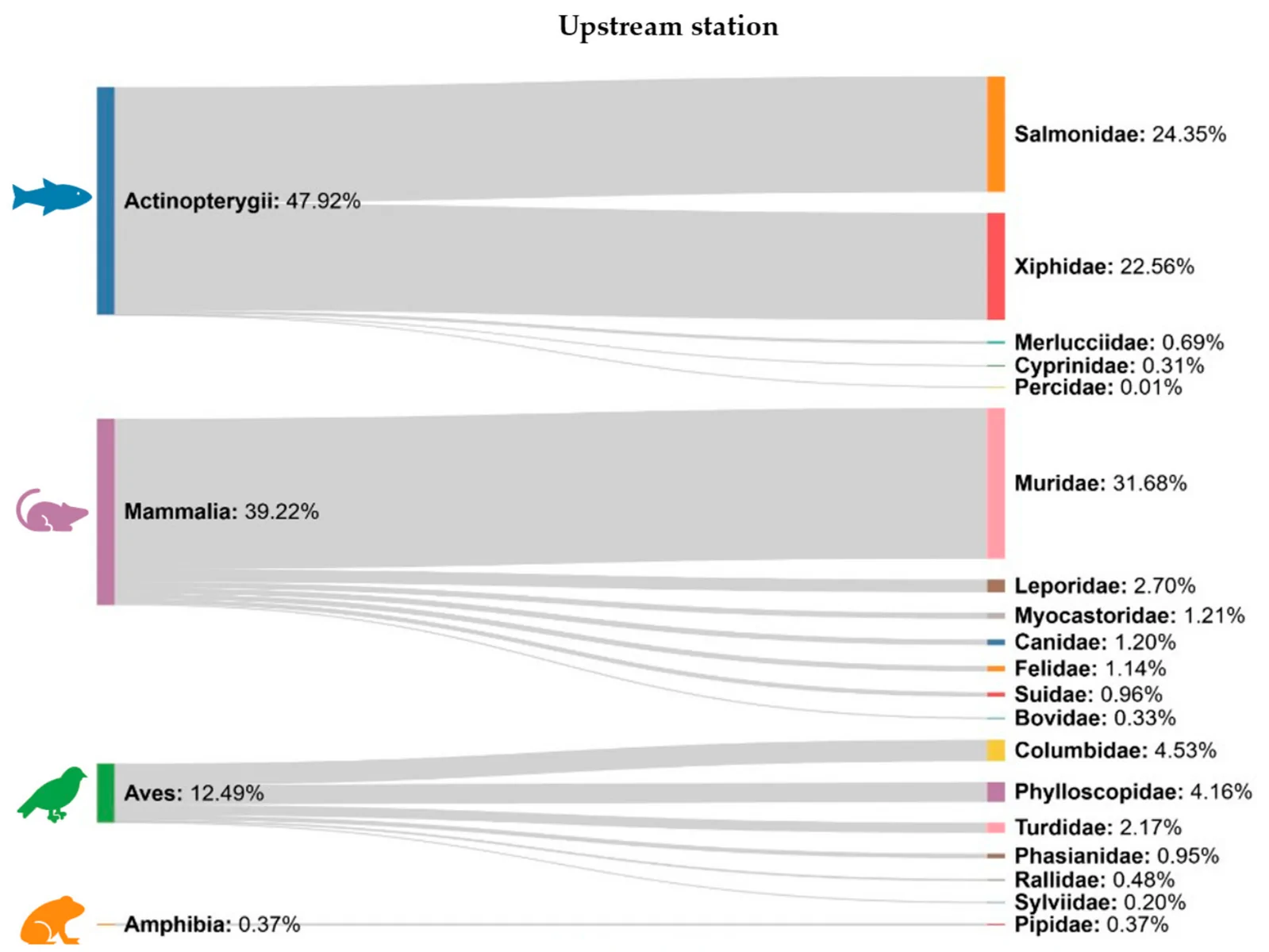
Taxonomic composition of the Upstream eDNA sample (representing pooled data from three sampling points, out of nine total sites sampled along the river). A hierarchical representation of the relative read abundances (expressed as percentages) of the detected classes and their respective families. Flow band thickness reflects the quantitative proportion of sequence reads for each taxon within the sample.

At the Upstream station, the total eDNA pool (186,334 curated reads) was distributed across four vertebrate classes. Actinopterygii dominated the sequence pool, accounting for 47.92% of the total signal (89,290 reads), followed by Mammalia at 39.22%. Aves contributed 12.49%, whereas Amphibia constituted only a marginal fraction of the dataset (0.37%) (Figure 2). Within Actinopterygii, the sequence pool was strongly dominated in read representation by Salmoniformes (Salmonidae), representing 24.35% of the total reads, followed by Istiophoriformes (Xiphidae) with 22.56%. In contrast, Cypriniformes (Cyprinidae), Gadiformes (Merlucciidae), and Perciformes (Percidae) were detected at considerably lower relative read abundances (RRA), each contributing less than 1% of the total signal. Among Mammalia, Rodentia overwhelmingly dominated the mammalian sequence pool, primarily due to the high proportion of reads assigned to Muridae (31.68% of all curated reads), alongside a minor contribution from Myocastoridae (1.21%). Additional mammalian taxa included the orders Lagomorpha (Leporidae; 2.70%), Carnivora (Canidae; 1.20%, and Felidae; 1.14%), and Artiodactyla (Suidae; 0.96%, and Bovidae; 0.33%). Avian diversity was primarily represented by Passeriformes, specifically the families Phylloscopidae (4.16%), Turdidae (2.17%), and Sylviidae (0.20%). Other detected avian orders included Columbiformes (Columbidae; 4.53%), Galliformes (Phasianidae; 0.95%), and Gruiformes (Rallidae; 0.48%). Finally, Amphibia was exclusively represented by the order Anura (Pipidae), detected at a low relative read abundance (0.37%).

**Figure 3:**
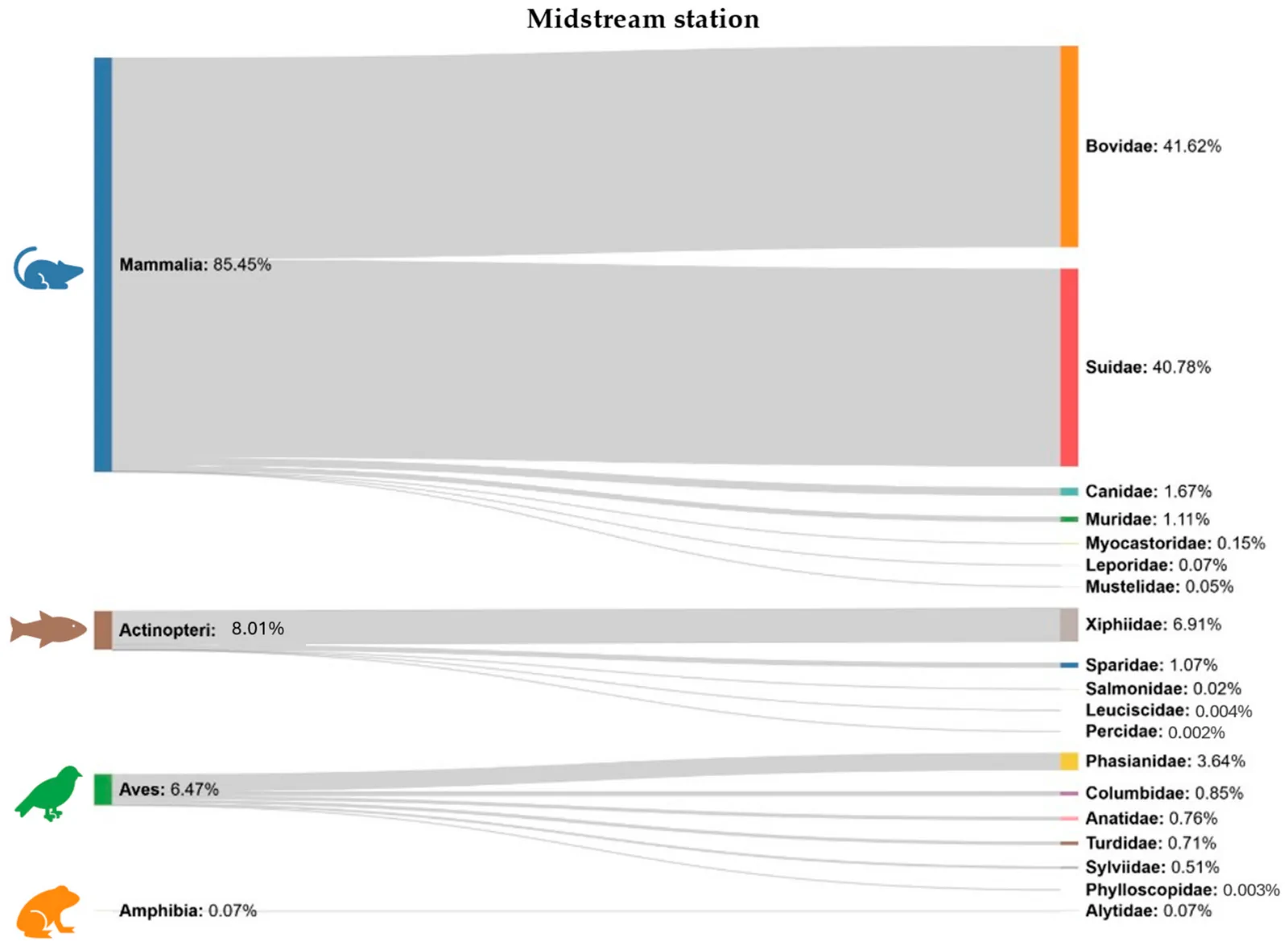
Taxonomic composition of the Midstream eDNA sample (representing pooled data from three sampling points, out of nine total sites sampled along the river). A hierarchical representation of the relative read abundances (expressed as percentages) of the detected classes and their respective families. Flow band thickness reflects the quantitative proportion of sequence reads for each taxon within the sample.

At Midstream station, the total eDNA pool (761,928 curated reads) was distributed across four vertebrate classes, with distinct compositional profiles. Mammalia heavily dominated the sequence pool, accounting for 85.45% of the total signal. Actinopterygii constituted the second highest read proportion at 8.01%, closely followed by Aves at 6.47%. Amphibia constituted only a negligible fraction of the dataset, representing 0.07% of the total sequence pool (Figure 3). Among Mammalia, the order Artiodactyla overwhelmingly dominated the sequence dataset, driven by the high read proportions of Bovidae (41.62%) and Suidae (40.78%), which collectively accounted for 82.40% of the total signal. Carnivora was the next highest mammalian eDNA signal, characterized by the detection of Canidae (1.67%) and a minor contribution from Mustelidae (0.05%). Rodentia comprised a small share of the mammalian signal, consisting of Muridae (1.11% total) and Myocastoridae (0.15%), while Lagomorpha (Leporidae) formed a minor component (0.07%). Within Actinopterygii, the ichthyological assemblage was strongly dominated in read representation by Istiophoriformes (Xiphidae), which alone accounted for 6.91%. Spariformes (Sparidae) constituted a secondary but notable component at 1.07%. In contrast, the remaining fish orders were detected at trace levels below 0.05% each, including Salmoniformes (Salmonidae; 0.02%), Cypriniformes (Leuciscidae; 0.004%), and Perciformes (Percidae; 0.002%). Avian diversity was predominantly represented by Galliformes (Phasianidae), which contributed 3.64% of the total signal. Passeriformes also provided a multi-familial signal, distributed across Turdidae (0.71%), Sylviidae (0.51%), and Phylloscopidae (0.003%). Other detected avian orders included Columbiformes (Columbidae; 0.85%) and Anseriformes (Anatidae; 0.76%). Finally, the class Amphibia was exclusively represented by the order Anura (Alytidae), which was detected at a low relative read abundance of 0.07%.

**Figure 4:**
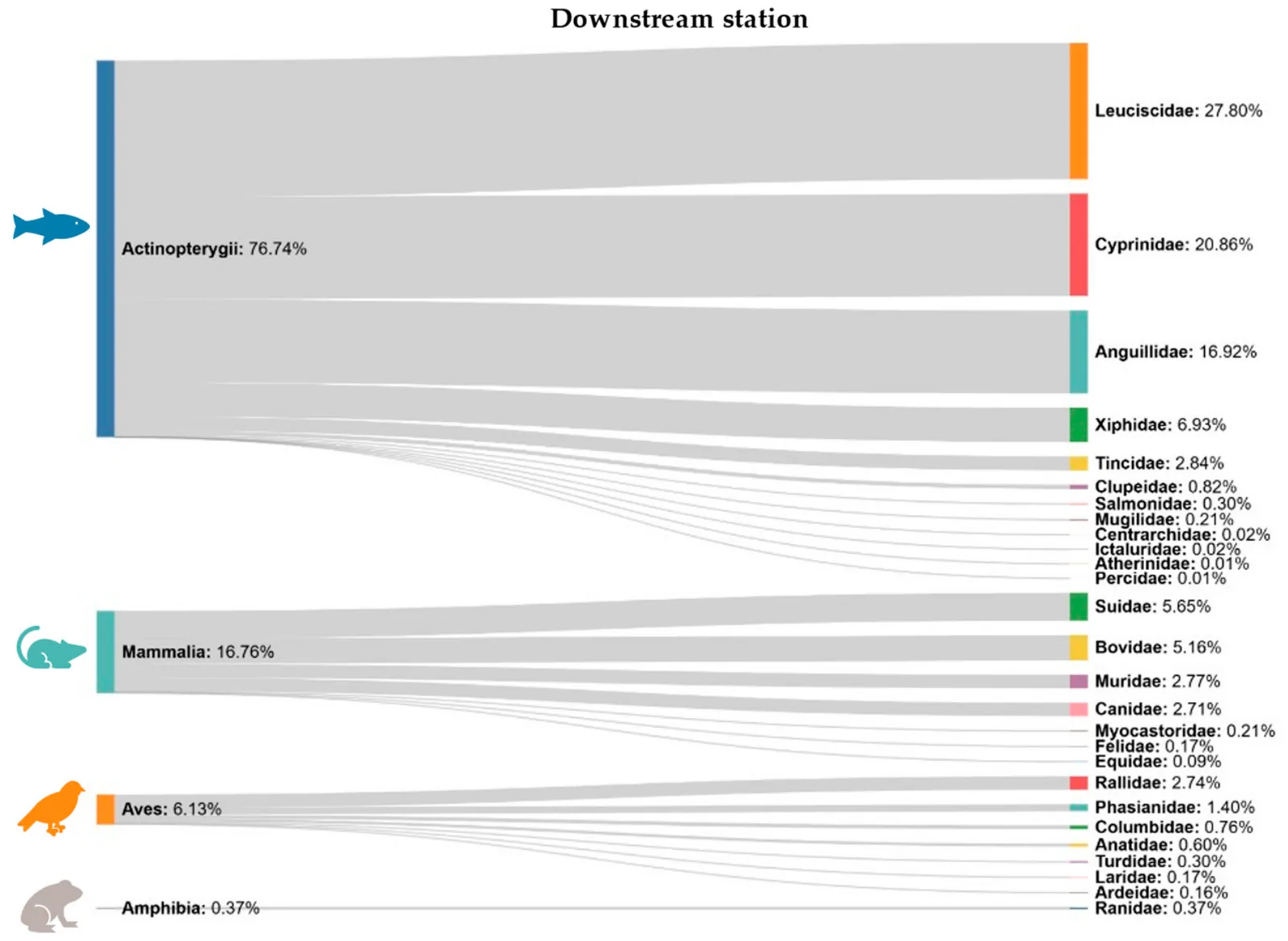
Taxonomic composition of the Downstream eDNA sample (representing pooled data from three sampling points, out of nine total sites sampled along the river). A hierarchical representation of the relative read abundances (expressed as percentages) of the detected classes and their respective families. Flow band thickness reflects the quantitative proportion of sequence reads for each taxon within the sample.

At the Downstream station, the total eDNA pool (357,649 curated reads) displayed a distinct compositional profile, heavily dominated by reads from aquatic taxa. Actinopterygii accounted for 76.74%. Mammalia accounted for the second largest read proportion at 16.76%, followed by Aves at 6.13%. Amphibia constituted a small fraction, representing 0.37% of the dataset (Figure 4). Within Actinopterygii, the order Cypriniformes represented the highest read proportion and taxonomic richness, collectively comprising 51.50% of the total dataset. This order was primarily represented by Leuciscidae (27.80%) and Cyprinidae (20.86%), with a minor contribution from Tincidae (2.84%). Anguilliformes (Anguillidae) also accounted for a substantial proportion of the ichthyological signal at 16.92%, followed by Istiophoriformes (Xiphidae) at 6.93%. Other fish orders were detected at lower sequence representations (<1%), including Clupeiformes (Clupeidae; 0.82%), Salmoniformes (Salmonidae; 0.30%), and Mugiliformes (Mugilidae; 0.21%). Trace signals below 0.05% each were recorded for Siluriformes (Ictaluridae; 0.02%), Centrarchiformes (Centrarchidae; 0.02%), Atheriniformes (Atherinidae; 0.01%), and Perciformes (Percidae; 0.01%). The mammalian fraction was predominantly shaped by the order Artiodactyla, which accounted for 10.81%, split between Suidae (5.65%) and Bovidae (5.16%). Rodentia constituted 2.98% of the total reads, mainly represented by Muridae (2.77%) alongside a minor presence of Myocastoridae (0.21%). Carnivora also contributed a noticeable share (2.88%), driven by Canidae (2.71%) and Felidae (0.17%). Perissodactyla (Equidae) represented a minor component, accounting for just 0.09%. Avian diversity was primarily characterized by the order Gruiformes (Rallidae), which contributed 2.74% of the total signal. Galliformes (Phasianidae) formed the second largest avian group at 1.40%. The remaining avian orders were detected at lower sequence representations, including Columbiformes (Columbidae; 0.76%), Anseriformes (Anatidae; 0.60%), Passeriformes (Turdidae; 0.30%), Charadriiformes (Laridae; 0.17%), and Pelecaniformes (Ardeidae; 0.16%). Finally, the class Amphibia was exclusively represented by the order Anura (Ranidae), which accounted for 0.37% of the overall dataset, indicating a minor but persistent amphibian eDNA presence in the downstream reach.

**Figure 5:**
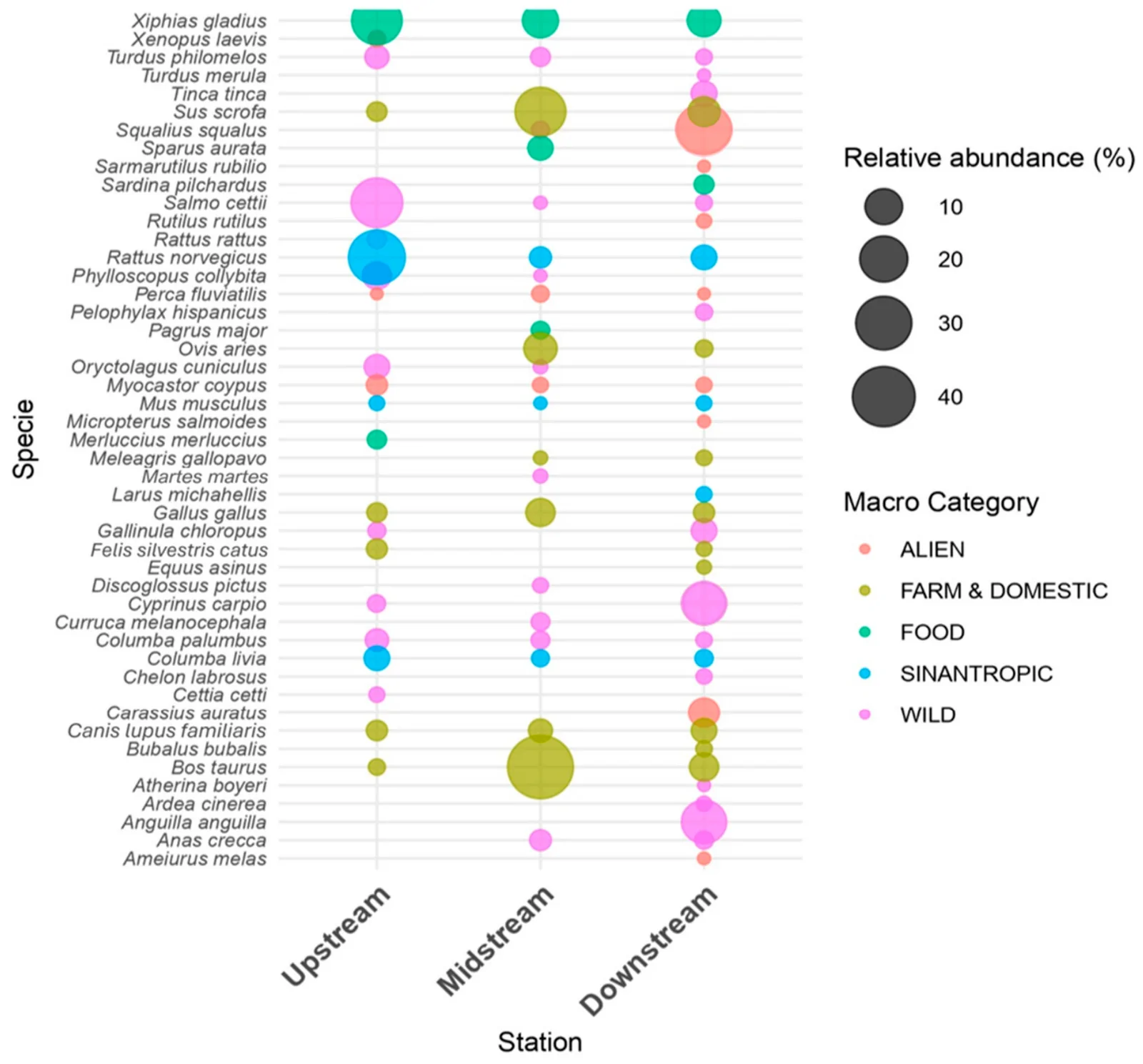
Taxonomic composition in the Oreto River. Bubble plot illustrating the relative read abundance (RRA, %) of vertebrate taxa detected via eDNA metabarcoding across the Upstream, Midstream, and Downstream sampling stations. Data for each station represent pooled eDNA sequence profiles from three sampling points (nine sampling points in total along the river gradient). Taxa (y-axis) are grouped into five a priori ecological/functional macro-categories (color-coded).

At the species level, the Upstream station revealed a vertebrate assemblage characterized by both freshwater-associated and terrestrial taxa (Figure 5). Among fish, *Salmo cettii* represented the freshwater species with the highest relative read abundance (24.35%), whereas *Xiphias gladius* showed an extremely strong eDNA signal (22.56% of total reads). Other fish species, including *Cyprinus carpio*, *Perca fluviatilis*, and *Merluccius merluccius*, were detected at comparatively lower eDNA signal intensities. Amphibians were represented exclusively by the invasive African clawed frog *Xenopus laevis* (0.37%). The avian sequence pool was composed primarily of passerine species, particularly *Phylloscopus collybita* (4.16%) and *Turdus philomelos* (2.17%). Riparian-associated taxa such as *Gallinula chloropus* were also detected (0.48%). Columbiformes were represented by both *Columba palumbus* (1.87%) and *Columba livia* (2.66%), while the domestic species *Gallus gallus* accounted for 0.95% of the dataset. *Cettia cetti* was detected at a lower read proportion (0.20%). Mammalian eDNA diversity was strongly dominated in sequence counts by rodents, particularly *Rattus norvegicus*, which represented the terrestrial species with the highest relative read abundance at the upstream station (30.83%). *Rattus rattus* and *Mus musculus* were also identified, although at substantially lower sequence representations (0.71% and 0.14%, respectively). Additional mammalian taxa included *Oryctolagus cuniculus* (2.70%), *Myocastor coypus* (1.21%), *Canis lupus familiaris* (1.20%), *Felis silvestris catus* (1.14%), *Sus scrofa* (0.96%), and *Bos taurus* (0.33%).

At the species level in the Midstream station, *Sus scrofa* yielded the highest relative read abundance overall (40.78%), followed by *Bos taurus* (34.75%). *Ovis aries* also contributed substantially to the sequence pool (6.87%). Other mammalian species were detected at markedly lower eDNA signal intensities, including *Canis lupus familiaris* (1.67%), *Rattus norvegicus* (1.09%), *Myocastor coypus* (0.15%), *Oryctolagus cuniculus* (0.07%), *Martes martes* (0.05%), and *Mus musculus* (0.02%). Among fish taxa, *Xiphias gladius* generated the strongest fish-associated eDNA signal (6.91%). Other fish species were represented at substantially lower levels, including *Sparus aurata* (0.50%), *Pagrus major* (0.57%), *Squalius squalus* (0.004%), and *Perca fluviatilis* (0.002%), whereas *Salmo cettii* was detected only sporadically (0.02%). The avian assemblage was primarily characterized by sequence signals from domestic and synanthropic taxa. *Gallus gallus* represented the most prominent bird species in terms of read count (3.64%), followed by *Anas crecca* (0.76%), *Turdus philomelos* (0.71%), *Curruca melanocephala* (0.51%), *Columba palumbus* (0.53%), and *Columba livia* (0.32%). Additional avian taxa, including *Meleagris gallopavo* and *Phylloscopus collybita*, were detected only at low read proportions. Amphibians were represented exclusively by *Discoglossus pictus* (0.07%).

At the species level, the Downstream station exhibited the highest vertebrate diversity among all sampling sites, with a marked predominance of sequence signals from freshwater and estuarine-associated fish taxa. The fish sequence pool exhibited the highest read proportion in *Squalius squalus* (27.80%), followed by *Anguilla anguilla* (16.92%) and *Cyprinus carpio* (15.53%). Additional taxa generating relatively strong eDNA signals included *Xiphias gladius* (6.93%), *Carassius auratus* (5.33%), and *Tinca tinca* (2.84%). Other taxa, such as *Sardina pilchardus*, *Chelon labrosus*, *Atherina boyeri*, *Ameiurus melas*, *Micropterus salmoides*, and *Perca fluviatilis*, were detected at comparatively lower sequence representations. The endemic freshwater trout *Salmo cettii* was detected only sporadically (0.30%). Amphibians were represented exclusively by the Italian pool frog *Pelophylax* kl. *hispanicus* (0.37%). The avian sequence pool was dominated by riparian and aquatic-associated species, particularly *Gallinula chloropus*, which represented the strongest avian eDNA signal (2.74%). Other frequently detected species included *Gallus gallus* (1.16%), *Anas crecca* (0.60%), *Columba livia* (0.51%), *Turdus philomelos* (0.28%), and *Larus michahellis* (0.17%). Additional taxa, such as *Ardea cinerea*, *Meleagris gallopavo*, *Columba palumbus*, and *Turdus merula*, occurred only at relatively low sequence representations. Among mammals, *Sus scrofa* exhibited the highest read representation (5.65%), followed by *Bos taurus* (4.44%), *Canis lupus familiaris* (2.71%), and *Rattus norvegicus* (2.63%). Other mammalian taxa included *Ovis aries*, *Bubalus bubalis*, *Myocastor coypus*, *Felis silvestris catus*, *Mus musculus*, and *Equus asinus*, all detected at comparatively lower relative read abundances, reflecting the strong influence of both agricultural and anthropogenic activities within the downstream sector of the river.

**Figure 6:**
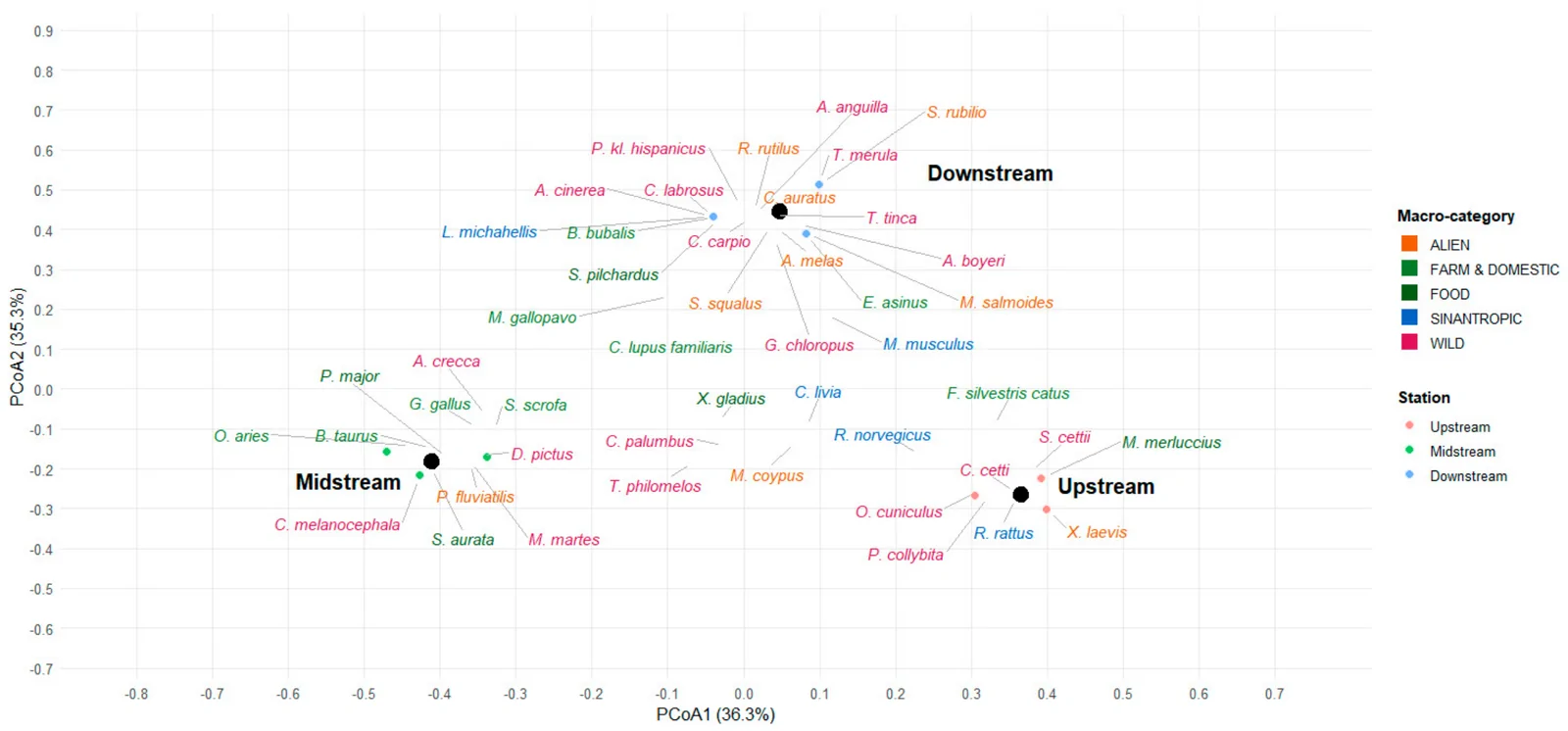
Principal Coordinate Analysis (PCoA) biplot (Bray–Curtis distance index) of vertebrate community composition along the Oreto River, based on the nine independent biological replicates (*n* = 3 per station: Upstream, Midstream, Downstream). Small points represent individual biological replicates, while large symbols indicate group centroids; colored text labels indicate projected taxa positions by ecological macro-category. Axes percentages show explained community variance (PCoA1: 36.3%; PCoA2: 35.3%). Grey segments connect high-density labels to their exact ordination coordinates.

Principal Coordinate Analysis (PCoA), performed on the replicate-level community data, revealed a clear spatial separation of vertebrate communities along the river gradient, with the first two axes explaining 36.3% and 35.3% of the total variance, respectively (cumulative variance = 71.6%) (Figure 6). PERMANOVA confirmed statistically significant differences in community composition among the three sampling stations (*pseudo-F* = 7.05, $R^{2}=0.70$, p=0.002), while multivariate dispersion analysis showed no significant differences in within-group variability among sites (F=0.64, p=0.619).

## 4. Discussion

Although freshwater ecosystems in the Mediterranean Basin host a biodiversity of high conservation value, they are severely threatened by anthropogenic pressures and remain among the least monitored environments; there are significant knowledge gaps regarding their biological composition and ecological status [33,34,35,36,37]. In this context, environmental DNA (eDNA) metabarcoding has consolidated into a powerful, non-invasive biomonitoring approach for watershed-scale assessments, enabling the rapid and sensitive characterization of biological communities through DNA transported along river networks [38,39,40].

In this study, eDNA metabarcoding provided an initial exploratory snapshot of vertebrate biological signatures across three representative sampling reaches of the Oreto River, an important refuge for Sicily’s freshwater fauna. To interpret the detected eDNA signals across the sampled reaches, taxa were evaluated according to five functional macro-categories (“Wild”, “Alien”, “Farm & Domestic”, “Synanthropic”, and “Food”) detailed in Table S2.

Furthermore, a qualitative comparison between our molecular inventory and historical conventional surveys (dating back to 2018) [26] showed substantial consistency with the known local fauna. Although the temporal and methodological gap precludes a rigorous quantitative comparison, this concordance highlights the complementary value of eDNA for biodiversity monitoring (Table 1).

### 4.1. Upstream Reach

The upstream site revealed an ecological pattern characterized by a combination of native and conservation-relevant taxa, terrestrial and avian species associated with riparian corridors, and eDNA signals potentially reflecting anthropogenic activities and non-native introductions.

Within the “Wild” macro-category, the putative detection of the Mediterranean trout, *Salmo cettii* Rafinesque, 1810, is particularly relevant from a conservation perspective. As an endemic Italian salmonid severely impacted by habitat degradation and genetic introgression [41], it is classified as “Critically Endangered” in the Italian Red List [42]. In Sicily, the extant distribution of this salmonid has long been considered restricted to southeastern Hyblaean river basins [43]; thus, if confirmed, this detection would represent a novel record for northwestern Sicily. However, given the limited length of the 106-bp 12S marker, potential reference database misannotations, and the complex history of stocking and hybridization in *Salmo* lineages, this signal must be treated strictly as a putative eDNA detection requiring independent validation, preferably supported by additional sequence-similarity or phylogenetic assessment.

Additionally, the upstream assemblage included the common carp, *Cyprinus carpio* Linnaeus, 1758, a species historically introduced but considered para-autochthonous in Sicily because of its long-standing integration into local aquatic ecosystems [44]. Its detection is consistent with previous eDNA investigations in Sicily [45,46] and with traditional records from the Oreto basin [26] (Table 1).

The terrestrial and avian components further reflected broader ecological patterns tied to habitat heterogeneity and watershed connectivity along the riparian corridor. Detections of species such as *Cettia cetti* (Temminck, 1820), *Gallinula chloropus* (Linnaeus, 1758), *Phylloscopus collybita* (Vieillot, 1817), *Columba palumbus* Linnaeus, 1758, and *Turdus philomelos* C. L. Brehm, 1831 were consistent with both regional ornithological records and previous observations specifically from the Oreto basin [26,47,48,49,50]. Furthermore, the detection of the para-autochthonous European rabbit, *Oryctolagus cuniculus* (Linnaeus, 1758), further highlights the presence of terrestrial wildlife utilizing the riparian area [51,52,53,54].

Potential anthropogenic-associated signals were also detected at the upstream site. Synanthropic rodents and birds, including *Rattus norvegicus* (Berkenhout, 1769), *Rattus rattus* (Linnaeus, 1758), *Mus musculus* Linnaeus, 1758, and *Columba livia* Gmelin, 1789, reflect proximity to human settlements and infrastructure [49,55]. Likewise, detections of domestic and livestock species, including *Sus scrofa* Linnaeus, 1758, *Bos taurus* Linnaeus, 1758, *Gallus gallus* (Linnaeus, 1758), *Canis lupus familiaris* Linnaeus, 1758, and *Felis silvestris catus* Linnaeus, 1758, are compatible with the agricultural and domestic context surrounding the headwaters. While these findings primarily suggest potential surface runoff from nearby agricultural activities and urban areas, eDNA data alone are insufficient to pinpoint specific sources or transport mechanisms. Indeed, alternative pathways, such as direct animal access to the watercourse, upstream transport, localized sewage discharges, slaughterhouse residues, or food waste, represent other plausible sources that cannot be disentangled without dedicated physicochemical or land-use modeling.

Similarly, genetic material attributed to commercial marine fish species, such as *Xiphias gladius* Linnaeus, 1758 and *Merluccius merluccius* (Linnaeus, 1758), cannot be interpreted as evidence of resident freshwater fish fauna. However, it is not possible to unequivocally determine the origin of this detected genetic material; while such findings might suggest anthropogenic sources—such as food waste or urban wastewater discharges—these explanations remain hypothetical. Without targeted wastewater sampling, complementary chemical indicators, or molecular source-tracking analyses, it is impossible to rule out alternative technical artifacts, including tag jumping during sequencing, laboratory cross-contamination, or DNA traces in commercial reagents [56,57].

Finally, the upstream site also highlighted an ecological pattern of non-native aquatic vertebrate presence and potential expansion, evidenced by taxa such as the European perch, *Perca fluviatilis* Linnaeus, 1758, an invasive species established in Sicilian freshwaters through aquaculture and fisheries stocking [44,58,59,60], whose presence in the Oreto River is likely attributable to past fish stocking practices in the region’s water bodies. Among the other non-native species, the semi-aquatic coypu *Myocastor coypus* (Molina, 1782) and the African clawed frog *Xenopus laevis* (Daudin, 1802) were also detected. While *M. coypus* in Sicilian freshwater systems is documented only in restricted areas [61], *X. laevis* is a well-established expanding invasive species introduced in 2004 [62,63]. However, for both taxa, the lack of independent observational data for the specific study area necessitates a cautious interpretive approach. Indeed, although query coverage and identity relative to reference sequences are 100% (Table S3), this does not in itself prove the local presence of the species, particularly in the case of isolated detections lacking independent field confirmation. Such reports should therefore be regarded as putative eDNA detections and interpreted as screening signals requiring targeted on-site validation.

### 4.2. Midstream Reach

The Midstream station was characterized by a heterogeneous eDNA assemblage that included generalist and synanthropic fauna, taxa associated with agricultural and domestic contexts, and non-native species. These ecological patterns provide useful indications regarding the ecological and anthropogenic context surrounding this river section. However, they should not be interpreted as direct evidence of specific environmental pressures or ecological degradation, as the present study did not include direct physicochemical or habitat-quality measurements.

Within the “Wild” macro-category, the Mediterranean trout (*Salmo cettii*) showed only trace levels of detection compared to the upstream reach. As discussed above, this signal must be regarded strictly as a putative eDNA detection requiring independent field and molecular validation, supported by nuclear markers or diagnostic alignments.

The remaining terrestrial and semi-aquatic fauna reflected an ecological pattern dominated by adaptable species. Alongside previously detected taxa (*Oryctolagus cuniculus*), the dataset recorded the genetic signature of the European pine marten *Martes martes* (Linnaeus, 1758), a species widespread in Sicily. Its sightings have increased due to more intensive surveying efforts, and it is now known to commonly utilize fragmented agricultural mosaics and riparian corridors for movement and foraging [49]. Furthermore, the genetic signature of the painted frog *Discoglossus pictus* Otth, 1837 was recorded. Historical herpetological surveys in the Palermo province [64] report a widespread distribution of this species in the regional territory, supporting its ecological resilience in human-impacted landscapes. The detection of these species is consistent with the heterogeneous agricultural and riparian landscape surrounding the middle reach of the watercourse. However, while these detections may contribute to characterizing the broader ecological context of the site, their presence cannot be directly linked to habitat alteration based solely on eDNA data.

Among the wild avifauna, the dataset confirms several species already detected upstream (*Turdus philomelos*, *Phylloscopus collybita*, and *Columba palumbus*), together with the Sardinian warbler *Curruca melanocephala* (Gmelin, 1789), a widespread Sicilian taxon synonymized with *Sylvia melanocephala* [65]. Waterfowl presence is represented by the common teal *Anas crecca* Linnaeus, 1758, a migratory species exploiting slow-flowing, nutrient-rich river sections during seasonal movements [50] and currently classified as ‘Endangered’ (EN) in the Italian Red List of Vertebrates [42]. Notably, all these avian species were previously documented along the Oreto River by Guadagna (2018) [26] (Table 1), further supporting the use of aquatic eDNA metabarcoding as a complementary tool for detecting local bird taxa.

Potential anthropogenic inputs were suggested by the combined occurrence of synanthropic, food-related, and farm/domestic signals. Persistent detections of *Rattus norvegicus*, *Mus musculus*, and *Columba livia* are compatible with the presence of human settlements and associated infrastructure in the surrounding landscape [49,55]. Their occurrence may reflect the use of riparian habitats by synanthropic species, although the eDNA data cannot determine the mechanisms underlying these patterns.

The detection of marine commercial species assigned to the “Food” category, including *Xiphias gladius*, *Sparus aurata* Linnaeus, 1758, and *Pagrus major* (Temminck & Schlegel, 1843), provides another potential anthropogenic signal. These taxa are not expected to represent resident freshwater fauna, and their DNA likely originated from external inputs. However, attributing these signals specifically to wastewater discharges or domestic food waste remains hypothetical. In the absence of wastewater-specific sampling, complementary chemical indicators, or molecular source-tracking analyses, it is impossible to rule out alternative technical artifacts, such as tag jumping during sequencing, cross-contamination, or trace DNA in commercial reagents [56,57]. These results should therefore be interpreted strictly as potential anthropogenic inputs rather than evidence of confirmed wastewater or food-waste contamination.

Similarly, the “Farm & Domestic” macro-category represented a substantial component of the eDNA profile, including *Bos taurus*, *Sus scrofa*, *Ovis aries* Linnaeus, 1758, *Gallus gallus*, *Meleagris gallopavo* Linnaeus, 1758, and *Canis lupus familiaris*. Although these results may be consistent with surface runoff from the agricultural and pastoral areas surrounding the intermediate reach, livestock-associated DNA is not in itself sufficient to demonstrate the presence of agricultural runoff, wastewater discharge, or consequent ecosystem degradation. Alternative transport pathways—such as direct animal access to the watercourse, diffuse environmental inputs, passive waterborne transport, or food/slaughterhouse waste—represent equally plausible sources that cannot be distinguished based on the current dataset.

Non-native taxa represented a further component of the midstream assemblage. Among these, the Italian chub, *Squalius squalus* (Bonaparte, 1837), native to the Italian peninsula but allochthonous in Sicily, has been introduced into artificial reservoirs and inland waters of the island, including for recreational fishing [44]. The detection of *Perca fluviatilis* and the semi-aquatic coypu *Myocastor coypus* further indicates the occurrence of non-native taxa in the eDNA assemblage. For the latter species, as established for the upstream reach, manual sequence verification using the online NCBI BLASTn tool (https://blast.ncbi.nlm.nih.gov/Blast.cgi, accessed on 10 September 2026) against the NCBI nt database confirmed 100% query coverage and 100% identity, accompanied by a significant sequence identity gap (>6.5%) relative to non-target reference sequences (Table S3). Nevertheless, these findings should be regarded as screening signals requiring targeted field validation, as high sequence identity alone does not demonstrate local species occurrence.

Taken together, the midstream eDNA profile reveals a heterogeneous ecological pattern in which generalist, synanthropic, potentially agricultural-associated, and non-native taxa occur alongside species also detected at the upstream site. These patterns are broadly compatible with the mixed agricultural and peri-urban context of the middle reach, but they should not be interpreted as evidence of direct environmental degradation or a progressive ecological decline without independent data on physicochemical parameters, habitat structure, hydromorphology, and other indicators of ecological status. Instead, the present results highlight the potential of eDNA metabarcoding as a rapid screening tool for identifying taxa and potential anthropogenic-associated signals that can inform subsequent targeted surveys and integrated environmental assessments.

### 4.3. Downstream Reach

As the Oreto River approaches its mouth, the downstream site was characterized by an ecological pattern shaped by a heterogeneous assemblage including freshwater, estuarine, and marine-associated taxa, together with terrestrial and synanthropic species.

Within the “Wild” macro-category, the fish assemblage showed a marked change in composition compared with the upstream and midstream reaches, with the occurrence of taxa associated with estuarine and coastal environments. The Mediterranean trout, *Salmo cettii*, previously detected in the upstream and midstream reaches, was represented only by residual levels of genetic signal. Given the lack of independent records of this species in northwestern Sicily, this detection remains strictly putative and requires independent field and molecular validation. In contrast, the downstream reach was characterized by the detection of the European eel, *Anguilla anguilla* (Linnaeus, 1758), a native catadromous species whose life cycle involves migration between marine spawning areas and continental waters [66]. Currently classified as “Critically Endangered” on the IUCN Red List due to multiple anthropogenic pressures [66], its detection is particularly relevant as it is directly corroborated by previous traditional surveys at the Oreto River mouth [26] (Table 1) and aligns with regional records across Sicilian hydrographic basins [45,67,68]. The native estuarine component was further represented by euryhaline transitional species, including the thicklip grey mullet, *Chelon labrosus* (Risso, 1827), and the bigscale sand smelt, *Atherina boyeri* Risso, 1810, both characterized by broad salinity tolerance and known to occur in coastal lagoons, estuaries, and lower river sections. *A. boyeri* has previously been detected through eDNA in landlocked Sicilian reservoirs, including Lakes Poma, Scanzano, Rosamarina, and Garcia [45,46], and has also been documented through traditional surveys in the Dirillo and Irminio basins [67,68]. The presence of *C. labrosus* is likewise consistent with the environmental characteristics of lowland and estuarine habitats. However, because physicochemical parameters were not measured, the occurrence of these taxa cannot be used to infer a progressive deterioration in environmental quality. The fish assemblage also included the para-autochthonous *Cyprinus carpio*, previously detected upstream, and *Tinca tinca* (Linnaeus, 1758), both of which were also reported by Guadagna (2018) [26] (Table 1).

The herpetofaunal and avian assemblages further reflected the ecological heterogeneity of the lower river. The dataset detected the Italian pool frog, *Pelophylax* kl. *hispanicus* (Bonaparte, 1839), a water-dependent amphibian associated with permanent aquatic habitats [69]. This result provides a more resolved molecular assignment than the genus-level identification (*Pelophylax* spp.) previously reported by Guadagna (2018) [26] (Table 1), although independent validation would be desirable for a species-level interpretation. The avian assemblage was characterized by taxa associated with both riparian vegetation and open-water habitats. The common moorhen, *Gallinula chloropus*, previously detected upstream, occurred together with the grey heron, *Ardea cinerea* Linnaeus, 1758. As a strictly protected species under the EU Birds Directive (2009/147/EC) [70], *Ardea cinerea* acts as an apex predator in shallow waters, feeding on fish and amphibians. In Sicily, it is primarily a wintering and migratory species, with rare reports of local nesting [49]. Its presence may be associated with that of mugilids and cyprinids, which congregate in the slow-flowing waters of the downstream section. Additional avian taxa included *Columba palumbus*, *Turdus philomelos*, and the newly detected common blackbird, *Turdus merula* Linnaeus, 1758; all these avian species were previously documented along the Oreto River by Guadagna (2018) [26] (Table 1), further confirming the utility of aquatic eDNA metabarcoding as a complementary tool for local avian monitoring.

The “Farm & Domestic” macro-category remained an important component of the downstream eDNA profile. Signals assigned to *Bos taurus*, *Sus scrofa*, *Ovis aries*, *Gallus gallus*, *Meleagris gallopavo*, together with *Bubalus bubalis* (Linnaeus, 1758) and *Equus asinus* Linnaeus, 1758, may once again reflect the agricultural, rural, and urban settings found within the basin. However, these detections cannot be interpreted as evidence that runoff from grazing or agricultural areas affects the downstream reach. Indeed, as previously mentioned, the detected DNA can reach the site via multiple alternative pathways—such as direct animal access, diffuse transport, or hydrological connectivity—without the eDNA data alone making it possible to determine a specific mechanism. Similarly, detections of *Canis lupus familiaris* and *Felis silvestris catus* were consistent with residential and urban settings but cannot be used to infer ecosystem degradation or assess the site’s ecological status.

The “Synanthropic” macro-category provided additional evidence of taxa associated with human-modified environments. The detection of *Rattus norvegicus*, *Mus musculus*, and *Columba livia* is consistent with the urban and peri-urban context of the lower river. The yellow-legged gull, *Larus michahellis* Naumann, 1840, was also detected; this opportunistic species is commonly associated with coastal and urban environments, and its occurrence in the lower Oreto has previously been documented [26] (Table 1). Together, these taxa characterize the anthropized context of the lower river without providing a standalone quantitative measure of specific environmental pressure. In addition, the downstream reach also captured a signal of marine commercial species, grouped under the “Food” macro-category, including the swordfish, *Xiphias gladius*, and the European pilchard, *Sardina pilchardus* (Walbaum, 1792). Consistent with upstream and midstream observations, these strictly marine taxa do not represent resident freshwater fauna. While such findings might suggest anthropogenic sources—such as food waste or municipal wastewater discharges—these explanations remain hypothetical. In the absence of targeted wastewater sampling, complementary chemical indicators, or molecular source-tracking analyses, it is impossible to exclude technical artifacts such as tag jumping or laboratory cross-contamination [56,57]. These detections must therefore be interpreted strictly as potential anthropogenic inputs rather than confirmed wastewater or food-waste contamination.

Finally, the lower course featured several taxa clustering under the “Alien” macro-category. The ichthyofauna was dominated by the allochthonous Italian chub *Squalius squalus*, whose presence in Sicily is linked to historical introduction events [71] and has been previously documented through regional eDNA surveys [45]. In addition, the community includes the recurrent detection of *Perca fluviatilis*, together with non-native cyprinids such as *Sarmarutilus rubilio* (Bonaparte, 1837) and *Rutilus rutilus* (Linnaeus, 1758), which represents a widespread Eurasian species translocated into several Mediterranean freshwater systems that can establish in lentic and lowland river habitats. While several of these taxa are native to mainland Italy, they are all considered allochthonous within Sicilian inland waters [67,72,73], reflecting repeated human-mediated introductions into reservoirs and fluvial networks. The non-native community was completed by other generalist fishes, including *Carassius auratus* (Linnaeus, 1758), an ornamental Asian species often released into the wild [74], the black bullhead, *Ameiurus melas* (Rafinesque, 1820), naturalized in numerous Sicilian freshwater environments due to its high tolerance of variable environmental conditions [44] and the largemouth black bass, *Micropterus salmoides* (Lacepède, 1802), a North American predatory species introduced for sport fishing, frequently reported in Sicilian inland waters [44]. The composition of this assemblage is compatible with the long history of fish introductions and translocations in Sicilian inland waters. However, eDNA data cannot distinguish between direct stocking, secondary dispersal from upstream populations, or transport from connected lentic systems, and the contribution of these pathways therefore remains uncertain.

Among non-native species, detections of *Myocastor coypus* and *Xenopus laevis* were also registered. As discussed for the upstream and midstream reaches, although manual sequence verification using the online NCBI BLASTn tool (https://blast.ncbi.nlm.nih.gov/Blast.cgi, accessed on 10 September 2026) confirmed 100% sequence identity to reference databases (Table S3), metabarcoding signals alone cannot prove physical local establishment, and these records must be regarded as putative screening signals requiring targeted field validation.

Overall, the downstream eDNA profile revealed a heterogeneous ecological pattern characterized by the coexistence of freshwater, estuarine, coastal, terrestrial, synanthropic, and non-native taxa. The occurrence of *Anguilla anguilla*, *Chelon labrosus*, and *Atherina boyeri* is fully consistent with the transitional nature of the lower river and its connection with the coastal environment, while terrestrial and synanthropic components reflect the heterogeneous landscape surrounding the river mouth. However, these ecological patterns should be interpreted as evidence of community composition and environmental connectivity rather than as direct evidence of ecological condition. Integrating eDNA metabarcoding with physicochemical measurements, habitat assessment, and targeted field surveys remains essential to evaluate the ecological condition of the downstream reach and distinguish natural environmental patterns from specific anthropogenic pressures.

### 4.4. Limitations

Despite the significance of the results obtained in this study, which demonstrate the effectiveness of environmental DNA (eDNA) metabarcoding as a non-invasive tool for assessing aquatic biodiversity, several methodological limitations must be explicitly acknowledged.

Firstly, spatial and temporal replicability was limited, as sampling was restricted to three representative river stations during a single campaign in October 2023. Consequently, the results offer only a preliminary, exploratory view of vertebrate composition at these locations. Indeed, a single-campaign design limits the ability to distinguish between persistent spatial patterns, seasonal variability, or transient disturbance events. Accordingly, differences among stations should be interpreted as site-specific patterns reflecting the conditions prevailing at the time of sampling, rather than as evidence of persistent ecological gradients across the river basin.

Second, both molecular and environmental processes may influence the observed taxonomic composition. Primer-template mismatches and taxon-specific PCR amplification efficiencies can result in differential detection, taxonomic dropout, or preferential amplification, thereby affecting the representation of taxa in the final dataset. In addition, eDNA transport within river networks, including downstream advection and inputs from lateral runoff or adjacent terrestrial environments, may lead to the detection of DNA originating outside the immediate sampling area. Relative read abundance (RRA) should therefore be interpreted as a semi-quantitative indicator of the relative molecular signal within the dataset, rather than as a direct measure of absolute species abundance, population density, or biomass.

Third, bioinformatic processing relied on a standardized workflow implemented in QIIME 1.9.1, using open-reference OTU clustering at a 97% sequence similarity threshold, the RDP Classifier v.2.2, and reference databases compiled from NCBI and MitoFish records accessed in 2020–2021. Although the workflow was established during the initial processing of the data in late 2023, more recent approaches, including amplicon sequence variant (ASV) inference using QIIME 2 and DADA2, together with updated reference databases, could improve sequence resolution and refine taxonomic assignments. However, the extent to which these methodological updates would affect the present results cannot be established without reprocessing the dataset. The taxonomic inventory and ecological patterns reported here should therefore be interpreted in light of the analytical framework and reference resources used.

A further important limitation of this study concerns the taxonomic resolution achievable with the 12S marker used for eDNA metabarcoding. The amplified fragment is relatively short (~106 bp), a feature that improves amplification success from environmentally degraded DNA but simultaneously limits the amount of phylogenetically informative variation available for species discrimination. The 97% sequence identity threshold used during OTU picking was applied exclusively for clustering reads against reference OTUs and should not be interpreted as evidence supporting species-level identification. Similarly, the confidence value generated by the RDP classifier reflects statistical support for a taxonomic assignment within the reference database but does not necessarily guarantee that closely related species can be unambiguously differentiated. For several vertebrate groups, multiple congeners are known to share identical or nearly identical haplotypes within the 12S-V5 region. In such cases, assignment to a single species may not be possible based solely on the metabarcoding marker, even when a high classification confidence score is obtained. Therefore, species-level detections should be interpreted as the most likely taxonomic assignment given the available sequence information and current reference database rather than definitive evidence for the presence of a particular species. Nevertheless, these limitations do not affect the principal ecological conclusions of the study, which rely on broader patterns of vertebrate community composition and occupancy rather than on unequivocal discrimination among closely related taxa.

Finally, the absence of concurrent physicochemical measurements, systematic habitat assessments, and multi-season monitoring limits the extent to which the observed eDNA patterns can be related to specific environmental pressures, habitat degradation, or water-quality conditions. Although eDNA metabarcoding provides valuable information on vertebrate DNA occurrence, it cannot independently establish causal relationships between environmental conditions and community composition or determine the demographic status of detected populations. Isolated detections and records outside known geographic ranges, particularly those involving invasive or conservation-relevant taxa, should therefore be treated as preliminary screening signals requiring further investigation rather than as conclusive evidence of local occurrence. Future monitoring of the Oreto River should combine broader spatial coverage and seasonal replication with complementary genetic markers, targeted qPCR or ddPCR assays for critical detections, and conventional biological surveys. Integrating these approaches with physicochemical measurements and systematic habitat assessments would provide a stronger basis for validating species records, interpreting community patterns, and developing a more comprehensive assessment of biodiversity and ecological status across the catchment.

## 5. Conclusions

This study provides a baseline characterization of vertebrate biodiversity in the Oreto River using environmental DNA (eDNA) metabarcoding. Although constrained by a limited spatial (three sampling stations) and temporal (a single sampling campaign) scope, and subject to inherent methodological limitations, our findings demonstrate the high sensitivity of aquatic eDNA for biomonitoring heavily impacted Mediterranean freshwaters.

Key conservation findings include a putative detection of the Mediterranean trout (*Salmo cettii*)—requiring independent validation through traditional surveys and nuclear markers—alongside confirmed records of *Anguilla anguilla*, *Ardea cinerea*, and *Anas crecca*.

Detections of terrestrial mammals and avian species highlighted broader ecological patterns of connectivity linking the river to its surrounding riparian corridor. Simultaneously, signals from non-native, synanthropic, and domestic taxa reflected potential surrounding land use, while marine commercial fish eDNA suggested potential exogenous anthropogenic inputs (e.g., food waste) rather than confirmed wastewater discharges.

The observed ecological patterns, supported also by multivariate ordinations (PCoA) and permutation tests, indicate that the upstream reach appeared to function as a partial refuge for sensitive native fauna (despite early signals of human-associated taxa in the surroundings), the intermediate reach reflected the influence of surrounding agricultural and livestock activities, and the downstream estuarine site supported an altered community with a notable presence of non-native fish species. However, in the absence of direct physicochemical measurements, claims of a progressive downstream ecological degradation remain strictly hypothetical.

Overall, our results demonstrate that eDNA metabarcoding is an effective, sensitive, and non-invasive preliminary approach for assessing vertebrate assemblages in Mediterranean freshwater ecosystems. Its broad qualitative concordance with historical conventional surveys, combined with its ability to detect rare and elusive species as well as biological signatures of potential human activities, supports its integration into future biodiversity monitoring and conservation programs. To fully translate these preliminary insights into adaptive management strategies, future studies incorporating multi-seasonal sampling, direct water-quality monitoring, and an expanded spatial network across the entire catchment will be essential.

## Figures and Tables

**Table 1:** Comparison of vertebrate taxa detected via eDNA metabarcoding in the present study and traditional visual/capture surveys reported by Guadagna (2018) [26] for the Oreto River.

Class	Scientific Name	Present Study			Guadagna, 2018
		Upstream	Midstream	Downstream	
**Amphibia**	*Pelophylax* kl. *hispanicus* (Bonaparte, 1839)	-	-	X	X (as *Pelophylax* spp.)
**Actinopterygii**	*Anguilla anguilla* (Linnaeus, 1758)	-	-	X	X
	*Cyprinus carpio* Linnaeus, 1758	X	-	X	X
	*Tinca tinca* (Linnaeus, 1758)	-	-	X	X
**Aves**	*Anas crecca* Linnaeus, 1758	-	X	X	X
	*Ardea cinerea* Linnaeus, 1758	-	-	X	X
	*Cettia cetti* (Temminck, 1820)	X	-	-	X
	*Columba palumbus* Linnaeus, 1758	X	X	X	X
	*Curruca melanocephala* (Gmelin, 1789)	-	X	-	X (as *Sylvia melanocephala*)
	*Gallinula chloropus* (Linnaeus, 1758)	X	-	X	X
	*Larus michahellis* Naumann, 1840	-	-	X	X
	*Phylloscopus collybita* (Vieillot, 1817)	X	X	-	X
	*Turdus merula* Linnaeus, 1758	-	-	X	X
	*Turdus philomelos* C.L. Brehm, 1831	X	X	X	X

X indicates presence; - indicates absence.

## Data Availability

The original contributions presented in this study are included in the article/Supplementary Materials. Raw sequencing data supporting the findings of this study are available in the NCBI SRA repository under accession PRJNA1542808. Further inquiries can be directed to the corresponding authors.

## Supplementary Materials

The following supporting information can be downloaded at: https://www.mdpi.com/article/10.3390/biology15201874/s1, Table S1: Filtered representative sequences and taxonomic classification data. Table S2: Systematic checklist of detected vertebrate taxa (Phylum Chordata), functional categorization, and classification rationale; Table S3: Taxonomic resolution and sequence identity metrics for key and unexpected taxa identified via eDNA metabarcoding. Refs [75,76,77,78,79,80,81,82,83,84,85,86,87,88,89,90,91,92,93,94] are cited in the Supplementary Materials.
