# Supplementary material for: Contribution of eDNA Metabarcoding to Model Ecosystems: Case Study of Threatened Peri-Urban Mediterranean River
Source: Biology (Basel). 2026 Oct 17;15(20):1874. doi: 10.3390/biology15201874 (PMC13701746; doi:10.3390/biology15201874)
Supplement: Supplementary file 1 [file biology-15-01874-s001.zip › Supplementary Table S2.pdf]

Supplementary Text S2 and Table S2

Supplementary Text S2. Criteria for Ecological Macro-Categorization

To facilitate ecological interpretation, detected vertebrate taxa were assigned *a priori* to five functional macro-categories: Wild, Alien, Farm & Domestic, Synanthropic, and Food. Categorization was established based on native or introduced status in Sicily, habitat preferences, and primary ecological roles, informed by literature, global biodiversity databases, and regional fauna atlases. Potential category overlaps were resolved using explicit criteria: taxa with dual domestic/feral or wild/synanthropic lineages were categorized based on their primary ecological footprint in the catchment.

In detail:

1. "Wild": native species (autochthonous, including resident, migratory, and regular wintering taxa) and long-established para-autochthonous taxa (historically introduced prior to 1500 CE) fully integrated into natural ecological networks;
2. "Alien": non-native (allochthonous) species introduced into Sicilian inland waters;
3. "Farm & Domestic": managed livestock, poultry, or companion animals.
4. "Synanthropic": generalist or opportunistic species closely associated with human settlements, urban infrastructure, and anthropogenic resources; and
5. "Food": commercial marine species that cannot naturally inhabit freshwater lotic systems. Following recent urban eDNA frameworks [1], their recovery in inland waters is interpreted as a putative exogenous signal—representing hypothetical human dietary waste, domestic runoff, municipal wastewater, or potential sequencing artifacts—rather than local living fauna.

The complete classification of all detected taxa, alongside their functional micro-category, macro-category, and taxon-specific bibliographic rationale, is detailed in Table S1 below.

Table S2. Systematic checklist of detected vertebrate taxa (Phylum Chordata), functional categorization, and classification rationale.

Species	Micro_category	Macro_category	Rationale
Ameiurus melas (Rafinesque, 1820)	Freshwater fish	ALIEN	Native to North America [2]; intentionally introduced into inland waters in Sicily for recreational or ornamental purposes and is now established [3].
Anas crecca Linnaeus, 1758	Riparian birds	WILD	Extant (non-breeding) in Sicily; regular wintering visitor and passage migrant consistently documented across Sicilian inland wetlands and river corridors [2].
Anguilla anguilla (Linnaeus, 1758)	Freshwater fish	WILD	Autochthonous catadromous fish species; baseline native component of Mediterranean river networks and estuarine corridors in Sicily [2,4].
Ardea cinerea Linnaeus, 1758	Riparian birds	WILD	Extant in Sicily; regular wintering visitor and passage migrant consistently documented across Sicilian inland wetlands and river corridors [2].
Atherina boyeri Risso, 1810	Freshwater fish	WILD	Autochthonous euryhaline species entering lowland river stretches from estuarine and coastal habitats [4].
Bos taurus Linnaeus, 1758	Mammals	FARM & DOMESTIC	Domestic livestock species originating from the domestication of the wild aurochs *Bos primigenius* [5].

Bubalus bubalis (Linnaeus, 1758)	Mammals	FARM & DOMESTIC	Domestic livestock species originating from the domestication of the wild water buffalo *Bubalus arnee* [6].
Canis lupus familiaris Linnaeus, 1758	Mammals	FARM & DOMESTIC	Domestic companion animal derived from gray wolves (*Canis lupus*) [7].
Carassius auratus (Linnaeus, 1758)	Freshwater fish	ALIEN	Native to Asia; intentionally introduced into inland waters in Sicily for recreational or ornamental purposes and is now established [3,8].
Cettia cetti (Temminck, 1820)	Riparian birds	WILD	Native resident avian species strictly bound to dense Mediterranean riparian vegetation and scrub [2,9].
Chelon labrosus (Risso, 1827)	Freshwater fish	WILD	Autochthonous euryhaline fish inhabiting lower river reaches [4].
Columba livia Gmelin, 1789	Terrestrial birds	SYNANTHROPIC	Feral synanthropic population; native wild lineages in Sicily are largely replaced or introgressed by domestic/feral stocks tied to anthropogenic environments [9].
Columba palumbus Linnaeus, 1758	Terrestrial birds	WILD	Native wild avian species breeding and expanding across forested, rural, and semi-natural landscapes in Sicily [9].
Curruca melanocephala (Gmelin, 1789)	Terrestrial birds	WILD	Native resident bird species widely distributed and abundant across Mediterranean shrublands, orchards, and rural landscapes in Sicily, potentially supplemented by wintering individuals [2,9].
Cyprinus carpio Linnaeus, 1758	Freshwater fish	WILD	Historically introduced prior to 1500 CE from Eurasia; para-autochthonous species fully integrated into natural ecological networks and river systems in Sicily [3].
Discoglossus pictus Otth, 1837	Amphibians	WILD	Native amphibian species (West-Mediterranean sub-endemic); widespread across Sicilian freshwater habitats, riparian corridors, and shallow breeding ponds [10,11].
Equus asinus Linnaeus, 1758	Mammals	FARM & DOMESTIC	Domestic working animal/livestock species originating from the domestication of the African wild ass *Equus africanus* [12].
Felis silvestris catus Linnaeus, 1758	Mammals	FARM & DOMESTIC	Domestic companion animal (derived from *F. s. lybica*) associated with human habitations, rural farms, and peri-urban areas along the river corridor [13].
Gallinula chloropus (Linnaeus, 1758)	Riparian birds	WILD	Native resident avian species strictly bound to dense riparian and aquatic vegetation along inland wetlands [2].
Gallus gallus (Linnaeus, 1758)	Terrestrial birds	FARM & DOMESTIC	Domestic poultry derived from the wild red junglefowl; raised universally as the primary livestock species globally for animal protein production. The detected eDNA is interpreted as originating from agricultural runoff from local poultry farms [14].
Larus michahellis Naumann, 1840	Riparian birds	SYNANTHROPIC	Opportunistic seabird dependent on urban and riverine anthropogenic waste [15].
Martes martes (Linnaeus, 1758)	Mammals	WILD	Autochthonous species present in Sicily since the Late Pleistocene, utilizing streams and riparian vegetation as movement corridors and foraging grounds [9].
Meleagris gallopavo Linnaeus, 1758	Terrestrial birds	FARM & DOMESTIC	Domestic turkey derived from wild North American populations; widely reared globally as a major livestock poultry species for meat production. The detected eDNA is interpreted as originating from agricultural runoff from local poultry farming [16].

Merluccius merluccius (Linnaeus, 1758)	Marine fish	FOOD	Commercial marine fish officially designated for human consumption (EU Regulation No 1379/2013) [17]. The detected eDNA signal in freshwater reaches is interpreted as a putative exogenous anthropogenic input (*e.g.*, domestic dietary waste or municipal wastewater) rather than local living fauna.
Micropterus salmoides (Lacepède, 1802)	Freshwater fish	ALIEN	Native to North America; intentionally introduced into inland waters in Sicily for recreational or ornamental purposes and is now established [3].
Mus musculus Linnaeus, 1758	Mammals	SYNANTHROPIC	Commensal rodent strongly bound to human habitations, agricultural structures, and urban drainage infrastructure along river corridors [18].
Myocastor coypus (Molina, 1782)	Mammals	ALIEN	Native to South America (IUCN, 2024); intentionally introduced in Sicily and is now established [3].
Oryctolagus cuniculus (Linnaeus, 1758)	Mammals	WILD	Historically introduced prior to 1500 CE from the Iberian Peninsula; para-autochthonous species fully integrated into natural ecological networks in Sicily [19].
Ovis aries Linnaeus, 1758	Mammals	FARM & DOMESTIC	Domestic livestock species derived from the domestication of the wild mouflon *Ovis gmelinii* [20].
Pagrus major (Temminck & Schlegel, 1843)	Marine fish	FOOD	Commercial marine fish (native to the Indo-West Pacific) imported globally for human consumption (European Commission, 2024) [21]. The detected eDNA signal in freshwater reaches is interpreted as a putative exogenous anthropogenic input originating from dietary waste or municipal effluents.
Pelophylax kl. *hispanicus* (Bonaparte, 1839)	Amphibians	WILD	Autochthonous water frog of the southern *P. bergeri*/*P.* kl. *hispanicus* hybridogenetic system, featuring high evolutionary interest and conservation value [22].
Perca fluviatilis Linnaeus, 1758	Freshwater fish	ALIEN	Native to Eurasia; intentionally introduced into inland waters in Sicily for recreational or ornamental purposes and is now established [3,4].
Phylloscopus collybita (Vieillot, 1817)	Terrestrial birds	WILD	Native resident and wintering avian species frequenting wooded riparian thickets and dense vegetation along river corridors [2].
Rattus norvegicus (Berkenhout, 1769)	Mammals	SYNANTHROPIC	Strictly anthropophilic rodent associated with urban settlements, sewer systems, and riverbanks, foraging on anthropogenic refuse and food waste [9,18].
Rattus rattus (Linnaeus, 1758)	Mammals	SYNANTHROPIC	Commensal rodent with high ecological plasticity, bound to human settlements, farm buildings, and modified rural habitats along river corridors [9,18].
Rutilus rutilus (Linnaeus, 1758)	Freshwater fish	ALIEN	Native to Eurasia; introduced into Sicilian inland waters where it is now established as an alien species [2,23].
Salmo cettii Rafinesque, 1810	Freshwater fish	WILD	Autochthonous Mediterranean salmonid [24].
Sardina pilchardus (Walbaum, 1792)	Marine fish	FOOD	Commercial pelagic marine fish designated for human consumption (EU Regulation No 1379/2013) [17]. The detected eDNA signal in freshwater reaches represents a putative exogenous anthropogenic input (*e.g.*, domestic dietary waste or municipal wastewater).
Sarmarutilus rubilio (Bonaparte, 1837)	Freshwater fish	ALIEN	Native to the Italian peninsula; introduced into Sicilian inland waters where it is now established as an alien species [25].

Sparus aurata Linnaeus, 1758	Marine fish	FOOD	Commercial marine fish widely traded for human consumption (EU Regulation No 1379/2013) [17]. The presence of eDNA in freshwater reaches is categorized as a hypothetical exogenous input originating from seafood refuse or municipal effluents.
Squalius squalus (Bonaparte, 1837)	Freshwater fish	ALIEN	Native to the Italian peninsula; introduced into Sicilian inland waters where it is now established as an alien species [23].
Sus scrofa Linnaeus, 1758	Mammals	FARM & DOMESTIC	Domestic livestock species derived from the wild boar; classified as "Farm & Domestic" based on the primary agricultural footprint of swine husbandry in the area, acknowledging potential overlap with wild or feral lineages [26].
Tinca tinca (Linnaeus, 1758)	Freshwater fish	WILD	Historically introduced prior to 1500 CE from Europe; para-autochthonous species fully integrated into natural ecological networks and river systems in Sicily [3].
Turdus merula Linnaeus, 1758	Terrestrial birds	WILD	Native resident bird species widely distributed and abundant across shrubby, forested, and agricultural landscapes in Sicily, supplemented by migratory wintering populations [2,9].
Turdus philomelos C.L. Brehm, 1831	Terrestrial birds	WILD	Extant (non-breeding) in Sicily; regular wintering visitor and passage migrant across Sicilian riparian woodlands, inland wetlands, and river corridors [2].
Xenopus laevis (Daudin, 1802)	Amphibians	ALIEN	Native to Africa [2]; it was intentionally introduced into inland waters in Sicily and is now established [3].
Xiphias gladius Linnaeus, 1758	Marine fish	FOOD	Oceanic pelagic marine fish of high commercial value for human consumption (EU Regulation No 1379/2013) [17]. The eDNA detection in freshwater reaches is framed as a potential exogenous anthropogenic input from dietary waste.

References

1. Stoeckle, M.Y.; Ausubel, J.H. Biomonitoring in the Anthropocene: Urban estuary environmental DNA tracks marine fish, terrestrial wildlife, and human diet. *PLoS ONE* **2026**, *21*, e0332676.
2. IUCN. The IUCN Red List of Threatened Species. Available online: https://www.iucnredlist.org/ (accessed on 27 September 2026).
3. Marrone, F.; Naselli-Flores, L. A review on the animal xenodiversity in Sicilian inland waters (Italy). *Adv. Oceanogr. Limnol.* **2015**, *6*, 2–12.
4. Battiata, M.; Termini, D.; Ferrito, V. The ichthyofauna of Mediterranean islands: A review of Sicilian river systems. *Research Square* **2026**, https://doi.org/10.21203/rs.3.rs-9071532/v1.
5. Loftus, R.T.; MacHugh, D.E.; Bradley, D.G.; Sharp, P.M.; Cunningham, P. Evidence for two independent domestications of cattle. *Proc. Natl. Acad. Sci. U.S.A.* **1994**, *91*, 2757–2761.
6. Minervino, A.H.H.; Zava, M.; Vecchio, D.; Borghese, A. *Bubalus bubalis*: A short story. *Front. Vet. Sci.* **2020**, *7*, 570413.
7. Castro-Fuentes, R.; Socas-Pérez, R. Unveiling the significance of dog domestication in cognitive dysfunction: Are wolves protected? *Open Vet. J.* **2025**, *15*, 6126.
8. Nocita, A.; Tricarico, E.; Bertolino, S. Fine-scale analysis of heavily invaded Italian freshwater fish assemblages. *Integr. Zool.* **2017**, *12*, 500–511.
9. ARPA Sicilia. *Atlante Della Biodiversità Della Sicilia: Vertebrati Terrestri*; Studi e Ricerche 6.; ARPA Sicilia: Palermo, Italy, 2008.

10. Stöck, M.; Grifoni, G.; Armor, N.; Scheidt, U.; Sicilia, A.; Novarini, N. On the origin of the recent herpetofauna of Sicily: Comparative phylogeography using homologous mitochondrial and nuclear genes. *Zool. Anz.* **2016**, *261*, 70–81.
11. Termine, R.; Turrisi, G.F. Amphibians and reptiles of the Nature Reserve of Pergusa Lake (Enna, Italy), emphasizing Sicilian endemisms. *Biodivers. J.* **2020**, *11*, 639–646.
12. Beja-Pereira, A.; England, P.R.; Ferrand, N.; Jordan, S.; Bakhiet, A.O.; Abdalla, M.A.; Luikart, G. African origins of the domestic donkey. *Science* **2004**, *304*, 1781.
13. Baca, M.; Popović, D.; Panagiotopoulou, H.; Marciszak, A.; Krajcarz, M.; Krajcarz, M.T.; Nadachowski, A. Human-mediated dispersal of cats in Neolithic Central Europe. *Heredity* **2018**, *121*, 557–563.
14. Lawler, A. *Why Did the Chicken Cross the World?* Atria Books: New York, NY, USA, 2014.
15. Ramos, R.; Ramírez, F.; Sanpera, C.; Jover, L.; Ruiz, X. Diet of yellow-legged gull (*Larus michahellis*) chicks along the Spanish Western Mediterranean coast: The relevance of refuse dumps. *J. Ornithol.* **2009**, *150*, 265–272.
16. Speller, C.F.; Kemp, B.M.; Wyatt, S.D.; Monroe, C.; Lipe, W.D.; Arndt, U.M.; Yang, D.Y. Ancient mitochondrial DNA analysis reveals complexity of indigenous North American turkey domestication. *Proc. Natl. Acad. Sci. U.S.A.* **2010**, *107*, 2807–2812.
17. European Union. Regulation (EU) No 1379/2013 of the European Parliament and of the Council of 11 December 2013 on the common organisation of the markets in fishery and aquaculture products. *Off. J. Eur. Union* **2013**, *L 354*, 1–21
18. Castillo, E.; Priotto, J.; Ambrosio, A.M.; Provensal, M.C.; Pini, N.; Morales, M.A.; Polop, J.J. Commensal and wild rodents in an urban area of Argentina. *Int. Biodeterior. Biodegrad.* **2003**, *52*, 135–141.
19. Lo Valvo, M.; La Scala, A.; Scalisi, M. Biometric characterisation and taxonomic considerations of European rabbit *Oryctolagus cuniculus* (Linnaeus, 1758) in Sicily (Italy). *World Rabbit Sci.* **2014**, *22*, 207–214.
20. Khamiruev, T.N. Evolution of the species *Ovis aries*. *Vavilov J. Genet. Breed.* **2026**, *30*, 696–707.
21. European Commission. EU Information System on Commercial Designations of Fishery and Aquaculture Products. Available online: https://fish-commercial-names.ec.europa.eu/ (accessed on 6 October 2026).
22. Bruni, A.; Razzetti, E.; Mirabella, I.; Mezzadri, S.; Ambrogio, A.; Faraone, F.P.; Corti, C.; Borghi, A.L.; Maglia, M.; Bellati, A. An updated assessment of alien water frog distribution in Italy. In *Proceedings of the SEH XX European Congress of Herpetology*, Milan, Italy, 2–6 September 2019; Poster.
23. Massimo, L.; Bernardi, F.; Carosi, A.; Ciuffardi, L.; Deoli, G.; Franzoi, P.; Zadra, M. The check-list of the Italian freshwater fish fauna. *Ital. J. Freshw. Ichthyol.* **2019**, *5*, 239–254.
24. Sanz, N.; Araguas, R.-M.; Giampiccolo, M.; Duchi, A. Native population structure beyond hatchery introgression in the endemic Sicilian trout. *Diversity* **2023**, *15*, 274.
25. Duchi, A. Nuove segnalazioni ed aggiornamento della distribuzione di rovella, *Sarmarutilus rubilio* (Bonaparte, 1837), in Sicilia. *Ital. J. Freshw. Ichthyol.* **2022**, *8*, 41–45.
26. Larson, G.; Dobney, K.; Albarella, U.; Fang, M.; Matisoo-Smith, E.; Robins, J.; Lowden, S.; Finlayson, H.; Brand, T.; Willerslev, E.; et al. Worldwide phylogeography of wild boar reveals multiple centers of pig domestication. *Science* **2005**, *307*, 1618–1621.
