# Supplementary material for: Contribution of eDNA Metabarcoding to Model Ecosystems: Case Study of Threatened Peri-Urban Mediterranean River
Source: Biology (Basel). 2026 Oct 17;15(20):1874. doi: 10.3390/biology15201874 (PMC13701746; doi:10.3390/biology15201874)
Supplement: Supplementary file 1 [file biology-15-01874-s001.zip › Supplementary Table S3.pdf]

Supplementary Table S3. Taxonomic resolution and sequence identity metrics for key and unexpected taxa identified via eDNA metabarcoding.

The table reports total read counts, sample distribution, query coverage (%), percentage identity to the top match, and percentage identity to the second-best match obtained via manual BLASTn searches against the NCBI nt database for the short 106-bp 12S fragment**.**

Taxon	Total Reads	Sample Distribution	Top BLASTn Match (Species)	Query Coverage (%)	Top Match Identity (%)	Second-Best BLASTn Match (Species)	Second-Best Identity (%)	Discriminatory Gap (%)
Myocastor coypus (Molina, 1782)	2255<br>1229<br>746	Upstream<br>Midstream<br>Downstream	*Myocastor coypus*	100%	100%	*Proechimys kulinae* da Silva, 1998	93.40%	6.60%
Xenopus laevis (Daudin, 1802)	692	Upstream	*Xenopus laevis*	100%	100%	N/A	N/A	N/A
